# Evaluating Honey Adulteration Through Physicochemical Characterization and Liquid Chromatography–Mass Spectrometry-Based Sugar Profiling

**DOI:** 10.3390/foods15061038

**Published:** 2026-03-16

**Authors:** Entesar Al-Hetlani, Bessy D’Cruz, Mohammed Hayssam, Bedraya Mandekar, Mohamed O. Amin

**Affiliations:** 1 Department of Chemistry, College of Science, Kuwait University, Sabah Al Salem University City, P.O. Box 5969, Safat 13060, Shadadiya, Kuwait; bessy.dcruz@ku.edu.kw (B.D.); mohamad1hayssam@gmail.com (M.H.); 2 Environmental & Life Science Research Center, Kuwait Institute for Scientific Research, P.O. Box 24885, Safat 13109, Shadadiya, Kuwait; bmandekar@kisr.edu.kw; 3 Department of Chemistry, University at Albany, SUNY, 1400 Washington Avenue, Albany, NY 12222, USA

**Keywords:** adulteration detection, Kuwaiti Sidr honey, syrup, LC-MS, fructose to glucose ratio, sugar profiling

## Abstract

The high market demand for Sidr honey, known for its nutritional and therapeutic properties, makes it susceptible to adulteration with sugar syrups, compromising authenticity and consumer safety. This study employed physicochemical tests and liquid chromatography–mass spectrometry (LC–MS) sugar profiling to analyze the impact of adulteration with corn, date, and agave syrups (5–35% *w*/*w*) on Kuwaiti *Ziziphus spina-christi* (Sidr) honey samples. Authentic Sidr honey exhibited pH values within 3.4–6.1, free acidity (FA) of <50 mEq kg^−1^, high electrical conductivity (mean EC: 1066.21 ± 353 µS cm^−1^), and moisture content <20%. Adulteration did not significantly affect pH or moisture (*p* > 0.05). FA significantly changed only in corn syrup-adulterated samples (*p* < 0.05). Electrical conductivity varied significantly with syrup type (*p* < 0.05). LC–MS was used to quantify the fructose (F) and glucose (G) contents, their ratio (F/G), and the total sugar content (F + G). For the authentic samples, F/G = 1.10–1.35, consistent with reported ranges. Corn syrup reduced F + G and F/G, date syrup raised both sugar contents, modestly changing F/G, while agave syrup, markedly increased both F/G and F + G. This integrated approach of physicochemical characterization and targeted sugar profiling effectively detects syrup adulteration, enhancing honey authentication, consumer protection, and market transparency.

## 1. Introduction

Honey is a natural food mainly comprising sugars, particularly fructose (F) and gluose (G), and traces of enzymes, amino and organic acids, vitamins, minerals, and volatile compounds. Rich in flavonoids and phenolic acids, honey’s potent antioxidant, antimicrobial, and anti-inflammatory properties make it valuable in both traditional and modern health practices [1,2]. *Ziziphus spina-christi* (Sidr) honey, produced from *Ziziphus* species, is a prime example of monofloral honey. Compared to polyfloral honeys, Sidr honey is highly valued and typically more costly because of its exceptional medicinal qualities [3]. Owing to its distinctive flavor, therapeutic benefits, nutritional value, and perceived purity, Sidr honey commands premium prices in both local and international markets. Sidr honey has gained considerable global recognition, ranking just behind Manuka honey in popularity. Closely related to *Ziziphus jujuba* (jujube) honey, Sidr honey is predominantly harvested in the arid and desert regions of Yemen, Saudi Arabia, Egypt, Libya, and Pakistan and holds considerable cultural and economic importance throughout the Middle East [4]. In Kuwait, honey production is primarily centered in agriculturally rich areas, such as Wafra in the south (S) and Abdali in the north (N), where Sidr honey is the main product, complemented by smaller seasonal harvests of eucalyptus and willow honeys. Therefore, ensuring Sidr honey’s authenticity is crucial to maintain consumer confidence and safeguard the reputation of both producers and the broader honey industry [5].

Honey adulteration continues to be an urgent global issue, often involving the addition of sugar syrups, such as regular and high-F corn, cane, or date syrups, to boost the honey’s volume or sweetness [2,6]. Such adulteration misleads consumers into viewing genuine honey as overpriced and may pose health risks as added substances can cause allergic reactions or toxicity [6,7]. Recent investigations have highlighted the global prevalence of honey adulteration. The Europol–INTERPOL OPSON X operation (2020–2021) found that 7% of honey samples were fraudulent, equivalent to 51,000 kg of adulterated honey, while the European Union “From the Hives” study reported 46% noncompliant samples out of 320 analyzed [8,9]. These fraudulent practices produce unfair competition in the food industry, particularly among beekeepers and honey producers, and may ultimately destabilize the market [10]. Despite international standards, such as those set by the Codex Alimentarius Commission, adulteration remains difficult to detect because the sugar profiles of commercial syrups often closely resemble those of natural genuine honey [2]. Elevated sucrose contents may indicate several types of adulteration, including the addition of low-cost sweeteners (such as cane or beet sugar), premature harvesting (which prevents the complete conversion from sucrose to G and F), or the artificial feeding of sucrose syrups to bees to boost the yield [11,12]. Additionally, honey can be adulterated by directly adding either F or G, disbalancing the natural fructose-to-glucose ratio (F/G) [13]. Another important indication is the hydroxymethylfurfural (HMF) content, which is often used to detect inverted-sugar-related adulteration [12,14]; however, heating or prolonged storage can also increase HMF contents, potentially leading to false positives in authenticity assessments [15].

To overcome these challenges, various analytical techniques, including isotope ratio analysis, chromatography, and spectroscopy, have been developed to detect honey adulteration. Although isotope analysis is effective for identifying C4 sugar adulterants, it has limited sensitivity for detecting plant-derived C3 sugars [16]. Chromatographic methods, such as high-performance anion-exchange chromatography (HPAEC), gas chromatography (GC), and high-performance liquid chromatography (HPLC), are widely employed to detect specific markers such as difructose anhydrides and other sugar derivatives [17,18,19,20,21]. However, advanced analytical tools, such as ultrahigh-performance liquid chromatography (UHPLC) coupled with quadrupole time-of-flight mass spectrometry (QTOF–MS), allow more precise profiling of sugar compositions and identification of adulterant-specific markers [22]. In addition, spectroscopic approaches, including infrared (IR), near-infrared (NIR), Raman, and nuclear magnetic resonance (NMR) spectroscopies are used for honey authentication. IR and NIR are inexpensive and rapid, while NMR is costly; Raman also provides useful insights, though all can be influenced by matrix interferants [23,24,25,26]. Among these methods, tandem liquid chromatography–mass spectrometry (LC–MS) is favored for honey authentication because it is a powerful confirmatory analytical technique with high sensitivity and selectivity. These characteristics enable reliable identification and quantification of multiple compounds within complex honey matrices, supporting accurate compositional profiling and the detection of potential adulteration [27,28]. Additionally, complementary physicochemical parameters, such as F/G, the total sugar content (F + G), pH, free acidity (FA), and electrical conductivity (EC), further support the detection of adulteration patterns [29].

To the best of our knowledge, this study presents the first comprehensive assessment of the effects of syrup adulteration on the sugar composition and physicochemical properties of Kuwaiti Sidr honey. Particularly, this work focuses on a commonly consumed honey with limited prior scientific characterization. Initially, the fructose (F) and glucose (G) contents of eleven authentic honey samples were analyzed to establish baseline values. Representative samples (S1 and S2 collected from Wafra in the south, and N1 from Abdali in the north) were then adulterated with corn, date, and agave syrups at concentrations ranging from 5% to 35% (*w*/*w*); these syrups were selected due to their widespread commercial use and their ability to closely mimic the physicochemical properties of honey. Physicochemical parameters (pH, free acidity, electrical conductivity, and moisture content) were determined using standard analytical methods, while sugar composition (F and G contents, F/G ratio, and total sugars, F + G) was quantified by LC–MS. This multiparameter analytical framework provides a robust approach for evaluating syrup-based adulteration and establishes previously unavailable baseline reference data for the authenticity assessment of Kuwaiti Sidr honey.

## 2. Materials and Methods

### 2.1. Chemical Materials and Standards

Analytical standards of glucose and fructose, sodium hydroxide (NaOH) pellets, acetonitrile, and ammonium hydroxide were obtained from Sigma-Aldrich Chemie GmbH (Schnelldorf, Germany). All the reagents were used as received, without further purification. Ultrapure deionized water (18.2 MΩ cm, PURELAB Chorus 1, ELGA, High Wycombe, UK) was used throughout the experiments.

### 2.2. Collection of Honey and Syrup Samples

Honey production in Kuwait is inherently limited due to its small geographic area, harsh desert climate, and restricted floral diversity, which constrain sample availability. All the honeys were produced during a single harvest season (November 2023 and February 2024), when Sidr trees (*Ziziphus spina-christi*) dominate nectar sources, resulting in naturally monofloral honey [30]. A total of eleven Kuwaiti Sidr honey samples were collected from two major agricultural regions in Kuwait and georeferenced using GPS coordinates: Abdili in the north (30.00° N, 47.73° E) and Wafra in the south (28.64° N, 47.93° E). In addition to two mixed-origin samples (obtained from random locations within Kuwait for which precise geographic information was not specified by the supplier). All honey samples were sourced directly from independent farms and verified through labelling and province information. For each sample lot, three jars were collected, totaling 33 jars. The Wafra samples were labeled S1 through S6; Abdili samples, N1 through N3; and mixed-origin samples, H1 and H2. For adulteration, corn, agave, and date syrups were sourced from a local supermarket and utilized as model adulterants. These syrups were selected for their widespread use as commercial sweeteners and ability to closely mimic the physicochemical properties of honey. Agave syrup, a C3 plant-derived sweetener, is particularly rich in F and exhibits one of the highest F/G values among those of natural sweeteners, making it a strong indicator of adulteration [31,32]. Corn syrup, a C4 plant-derived sweetener and the main source of G, is widely used in the food industry and alters the sugar balance of honey by increasing G [32,33]. Date syrup, which is abundant in the Middle East, possesses a sugar composition that closely matches that of honey, containing approximately 39.4% and 38.2% F and G, respectively, making its detection as an adulterant especially challenging [34]. These syrups’ prevalences and compositional similarities to honey underscore their relevance as adulterants in authenticity studies.

All the honey and adulterant samples remained in their original sealed containers and were stored in the dark at room temperature (~20–25 °C) until the analysis.

### 2.3. Preparation of Adulterated Honey Samples

Adulterated samples were prepared by blending authentic Sidr honey with corn, agave, or date syrup at final adulterant contents of 5%, 10%, 15%, 25%, and 35% (*w*/*w*) [35]. Adulteration levels were limited to a maximum of 35% because higher concentrations significantly alter the physicochemical characteristics of honey, making the mixture unrealistic and unrepresentative of common adulteration practices. Each blend had a total mass of 7.0 g. Prior to mixing; the honey was liquefied in a 40 °C water bath for 10 min and gently mixed for rehomogenization. Then, the honey and syrups were weighed on an analytical balance and combined by thorough manual stirring until homogeneous. Before the analysis, the mixtures were left to equilibrate to room temperature.

### 2.4. Analysis of Physicochemical Properties

Electrical conductivity (EC), pH, free acidity (FA) and moisture content were determined following International Honey Commission (IHC) methods [36]. The detailed physicochemical properties of authentic Kuwaiti Sidr honey have been previously reported in our earlier study on the physicochemical characteristics and trace metal contents of Kuwaiti Sidr honey [30]. Briefly, a 20% (*w*/*v*) solution was first prepared by dissolving 5.00 g of pure or adulterated honey in 25.0 mL of ultrapure water at 20 °C. Then, EC (µS cm^−1^) was measured using a previously calibrated digital conductivity meter (Orion Star A212, Thermo Scientific^TM^, Waltham, MA, USA).

Subsequently, ~5.00 g of each pure or adulterated honey sample was dissolved in 37.5 mL of ultrapure water and homogenized, and the solution pH was recorded at 20 °C using a calibrated pH meter equipped with a combined electrode (SevenCompact S220, Mettler Toledo, Greifensee, Switzerland).

Then, the same solutions used for measuring the pH were used for measuring the FA, which is defined as the amount of titratable acid in honey at the equivalence point [37]. The solutions were titrated using 0.1 M NaOH to pH 8.3 within 2 min, and the volume of NaOH used was recorded. FA was expressed as milliequivalents per kilogram of honey (mEq kg^−1^) and calculated using the following standard equation [38]:

(1) $$FA=\frac{volume\;of\;NaOH\;(mL)\;\times \;Normality\;of\;NaOH\;\times \;1000}{mass\;of\;the\;honey\;sample\;in\;Kg}$$

For moisture content measurement, ceramic crucibles were precleaned, dried in an oven at 105 °C for 1 h, cooled to room temperature in a desiccator, and weighed (W1). Approximately 1 g of honey was weighed in each crucible, and the crucibles were weighed again (W2). They were then heated in an oven (Unitemp, Harvard/LTE, Oldham, UK) at 105 °C for 4 h. After heating, the crucibles were cooled to room temperature in a desiccator and weighed as W3. The moisture content was calculated using the following equation [38]:

(2) $$Moisture\;content\;\%=\;\frac{W2-W3}{W2-W1}\times 100$$

For the measurement of EC, pH, FA and moisture content in authentic honey, three independent jars were analyzed, with each sample tested in triplicate. The reported values represent the mean across all jars. The same procedure was applied to the adulterated honey samples to ensure consistency.

### 2.5. LC–MS Analysis of Sugar Contents in Locally Produced Honeys

The sugar contents in the honeys, syrups, and honey–syrup blends were quantitatively analyzed using LC–MS (Thermo Scientific^TM^ Q Exactive^TM^ Focus quadrupole–orbitrap mass spectrometer, Thermo Fisher Scientific, Bremen, Germany). This instrument combines a quadrupole for ion selection and an orbitrap for high-resolution detection, making it highly effective for accurately identifying small molecules, such as G and F, which were selected as target analytes because they are the most abundant sugars in honey. The samples were prepared following a slightly modified version of a previously published protocol [33]. Briefly, 0.200 g of the sample was transferred to a 15 mL glass vial and dissolved in 2.00 mL of aqueous acetonitrile (1:1 *v*/*v*). Then, the mixture was vortexed for 5 min and passed through a 0.20 µm polytetrafluoroethylene (PTFE) syringe filter, and an aliquot of the filtrate (50 µL) was diluted with 450 µL of aqueous acetonitrile (1:1, *v*/*v*) immediately before injection in LC–MS, enabling the instrument to record consistent well-shaped spectral peaks and protecting the LC column from any contamination.

The honeys, syrups, and honey–syrup blends were analyzed using LC–MS operating in hydrophilic interaction liquid chromatography mode and equipped with an XBridge amide column (2.1 × 150 mm, 5 µm; Waters Corporation, Milford, MA, USA). The mobile phases were (A) 0.1% (*v*/*v*) aqueous ammonium hydroxide and (B) acetonitrile mixed with 0.1% (*v*/*v*) ammonium hydroxide. The gradient program was as follows: 0.0–1.0 min, 87.5% B; 1.0–4.0 min, linear to 70% B; 4.0–5.0 min, to 50% B; 5.0–7.0 min, to 20% B; 7.0–8.0 min, back to 85% B; hold at 85% B to 10.0 min for re-equilibration. The column temperature, flow rate, and injection volume were 60 °C, 0.60 mL min^−1^, and 1 µL, respectively.

The samples’ sugars were detected using MS, specifically, the orbitrap analyzer of the Q Exactive^TM^ Focus spectrophotometer, which offers high-resolution (e.g., a full width at half maximum of 70,000 at *m*/*z* 200) detection. The instrument was equipped with a heated electrospray ionization source and operated in negative ion mode, which is suitable for polar nonvolatile compounds, such as sugars. The samples were analyzed in the range *m*/*z* 100–1000. The typical source parameters were a spray voltage of 3 kV, a sheath gas flow rate of 30 arbitrary units (a.u.), an auxiliary gas flow rate of 14 a.u., a capillary temperature of 120 °C, and an auxiliary gas heater temperature of 300 °C. Data were acquired and processed using Trace Finder and Thermo Xcalibur 4.2 software, which was used to integrate the G and F peak areas in the chromatograms.

The sugar components’ contents were quantified based on external calibration using G and F standards prepared at different concentrations and injected in triplicate for LC-MS analysis. Calibration curves were constructed based on the linear regression of the peak areas plotted as functions of G and F concentrations. In the honey samples, G and F concentrations were determined based on the linear-fitting equations shown in Figure S1a,b, respectively. The limits of detection (LOD) and quantification (LOQ) were calculated using the following equations:

Limit of detection (LOD)= (3 × σ)/s(3)

Limit of quantitation (LOQ)= (10 × σ)/s(4)

where σ represents the standard deviation of the blank and s denotes the slope of the calibration curve.

For authentic honey, three independent jars were analyzed in triplicate, and the measurements were reported as mean values across the jars. The same workflow was applied to the adulterated samples, and the G and F concentrations were averaged across the replicates.

### 2.6. Statistical Analysis

All statistical analysis was performed using OriginPro (version 2025 OriginLab Corporation, Northampton, MA, USA). Statistical analyses were performed to assess whether differences among the measured parameters were statistically significant (*p* ≤ 0.05). One-sample *t*-tests were used to determine whether the mean values of the physicochemical properties and sugar composition in authentic honey samples differed significantly from their corresponding reference or expected values. To evaluate the effect of syrup adulteration on the mean values of physicochemical parameters and sugar profiles across different adulteration levels, one-way analysis of variance (ANOVA) was applied [39], followed by post hoc Tukey test. A *p*-value ≤ 0.05 was considered statistically significant. Effect size was expressed as eta- squared (η^2^) calculated as the ratio of between-group sum of squares to total sum of squares (SS between/SS total), representing the proportion of total variation in each parameter that is attributable to differences in adulteration level.

## 3. Results

### 3.1. Analysis of Physicochemical Properties of Adulterated Honey Samples

As the adulteration of authentic honey, through either dilution or blending with plant-based syrups, not only deceives consumers but can also pose health risks, ensuring honey quality and authenticity is essential for both consumer safety and market integrity. Among the various analytical approaches, physicochemical markers, such as pH, FA, EC and moisture content, are indicators of both honey purity and botanical origin. To evaluate the honey quality and detect potential adulteration, we compared the physicochemical profiles of authentic Kuwaiti Sidr honey with those of the honey–syrup mixtures prepared using date, corn, and agave syrups.

**Figure 1:**
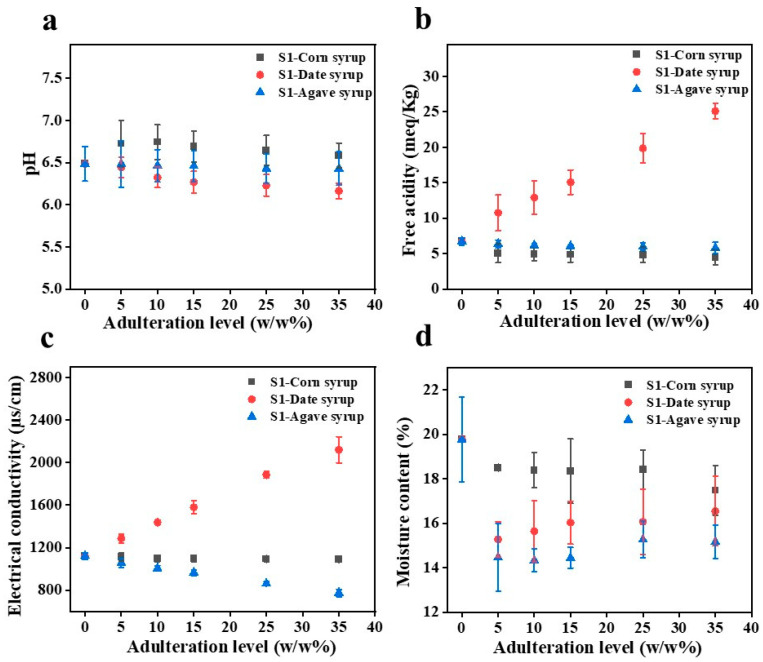
Evaluation of the physicochemical parameters of representative Kuwaiti Sidr honey sample S1 adulterated with corn, date, and agave syrups: (**a**) pH, (**b**) FA, (**c**) EC and (**d**) moisture content. Error bars represent one standard deviation.

Table S1 and Figure S2a–d represents the pH, FA, EC and moisture content values of the eleven authentic honey samples. The pH values ranged from 5.06 ± 0.01 (S2) to 6.49 ± 0.21 (S1), with a mean value of 5.77 ± 0.43, consistent with pH values previously reported for Sidr honey [40,41]. FA ranged from 6.75 ± 0.21 to 23.61 mEq kg^−1^ (for S1 and S6, respectively), with an average of 16.80 ± 4.57 mEq kg^−1^. The mean EC was 1066.21 ± 336.56 µS cm^−1^, where S5 and N3 (429.15 ± 13.93 and 1385.50 ± 139.30 µS cm^−1^) had the lowest and highest values, respectively. The moisture content of the Kuwaiti honey samples exhibited variation, ranging from 13.03 ± 1.13% (N1) to 19.77 ± 1.91% (S1), with a mean value of 15.60 ± 0.67%. To investigate the impacts of adulteration on the physicochemical properties, the changes in the pH, FA, EC and moisture content values of representative honey samples (S1, S2, and N1) adulterated with various concentrations of corn, date, and agave syrups were analyzed (Table S2). The variations in the pH, FA, EC and moisture content values for S1 and for S2 and N1 are presented in Figure 1a–d and Figure S3a–h, respectively.

Across all the honey samples, the pH levels remained relatively stable, only slightly varying depending on the adulterant type and concentration, as shown in Figure 1a and Figure S3a,b, respectively. In S1, the initial pH was 6.49 ± 0.21, and neither corn nor agave syrup noticeably changed the pH. At 5%, date syrup slightly decreased the pH from 6.45 ± 0.12 to 6.17 ± 0.09, suggesting mild acidification. The initial pH of S2 was lower (5.06 ± 0.01) than that of S1, and corn, date, and agave syrup adulterations slightly increased the pH. In N1, adulterations with 35% corn and date syrups slightly increased the pH to 5.74 and 5.82, respectively, while the pH did not measurably change when agave syrup was added. However, one-way ANOVA indicated that none of these changes were statistically significant (*p* > 0.05).

However, FA considerably varied depending on both the adulterant type and concentration, as presented in Figure 1b and Figure S3c,d, respectively. In S1, with increasing corn syrup content, FA consistently decreased from 6.75 ± 0.53 to 4.50 ± 1.06 mEq kg^−1^. In contrast, adulterations with 5% and 35% date syrup apparently increased FA from 10.78 ± 2.52 to 25.12 ± 1.06 mEq kg^−1^, respectively. Agave syrup minimally impacted the FA, which remained relatively stable between 6.37 ± 0.53 and 5.81 ± 0.80 mEq kg^−1^. In S2, corn syrup again substantially reduced the FA from 20.67 ± 2.20 to 11.87 ± 2.66 mEq kg^−1^. However, date syrup increased the FA, which peaked at 28.64 ± 0.00 mEq kg^−1^. Agave syrup gradually decreased FA from 14.20 ± 1.63 to 9.94 ± 0.86 mEq kg^−1^, while N1 followed a similar trend. Corn syrup reduced FA from 20.24 ± 2.65 to 11.87 ± 0.11 mEq kg^−1^, while date syrup progressively increased FA from 21.01 ± 0.59 to 32.69 ± 2.00 mEq kg^−1^. Agave syrup moderately decreased FA from 17.23 ± 2.12 to 11.90 ± 0.40 mEq kg^−1^. Nevertheless, the effects of date and agave syrup adulteration on FA were not statistically significant (*p* > 0.05), whereas FA differed significantly across corn syrup concentration levels (*p* < 0.05).

Across all the honey samples, corn, date, and agave syrup adulterations notably influenced EC (Figure 1c and Figure S3e,f, respectively). In S1, EC slightly decreased from 1121.50 ± 30.41 to 1090.00 ± 26.87 µS cm^−1^ with increasing corn syrup concentration. Agave syrup more markedly reduced EC from 1055.00 ± 38.18 to 774.35 ± 34.58 µS cm^−1^. Conversely, 35% date syrup adulteration substantially increased EC to 2119.50 ± 122.33 µS cm^−1^. In S2, corn syrup noticeably increased EC from 492.30 ± 3.39 to 679.10 ± 0.85 µS cm^−1^. Again, agave syrup decreased EC from 434.95 ± 3.32 to 325.10 ± 1.56 µS cm^−1^. Date syrup substantially elevated EC from 687.35 ± 28.35 to 1760.00 ± 50.91 µS cm^−1^. N1 followed a similar pattern. Corn syrup marginally increased EC from 1007.50 ± 31.82 to 1035.00 ± 28.28 µS cm^−1^, while agave syrup considerably decreased EC from 951.15 ± 43.77 to 696.80 ± 33.52 µS cm^−1^. Again, date syrup markedly increased EC from 1159.00 ± 36.77 to 2055.00 ± 33.94 µS cm^−1^. One-way ANOVA demonstrated that, at the 0.05 significance level, the population means of EC differed significantly across adulteration levels for all examined syrups (*p* < 0.05).

Additionally, the moisture content of honey samples adulterated with corn, date, and agave syrups was evaluated (Figure 1d and Figure S3g,h). Corn syrup exhibited a moisture content (19.82%) comparable to that of pure S1 honey, whereas agave and date syrups showed higher moisture contents of 21.72% and 24.21%, respectively. Consistent with these compositional differences, adulteration with corn syrup resulted in relatively stable moisture levels across all honey samples (Table S2). In contrast, adulteration with date and agave syrups led to gradual increases in moisture content with increasing adulteration levels. Specifically, for date syrup–adulterated honey, moisture content increased from 15.27 ± 0.71% to 16.55 ± 1.56% in S1, from 12.47 ± 3.58% to 15.79 ± 0.47% in S2, and from 13.95 ± 2.21% to 14.43 ± 2.97% in N1. Similarly, agave syrup adulteration increased moisture content from 14.47 ± 1.53% to 15.17 ± 0.74% in S1, from 14.20 ± 0.06% to 16.70 ± 0.17% in S2, and from 13.70 ± 2.41% to 14.84 ± 0.07% in N1. However, one-way ANOVA test indicated that these variations were not statistically significant (*p* > 0.05), demonstrating that moisture content did not differ significantly across adulteration levels for any of the syrups examined.

### 3.2. LC–MS Analysis of Honeys’ Sugar Contents

Sugars are the main constituents of honey, with F and G making up the largest proportion. As the F and G concentrations and relative balance are primarily based on honey’s geographical and floral sources [42], sugar profiles are important for determining honey quality, authenticity, and botanical origin. Because F and G are the predominant constituents relevant for characterizing the natural composition of honey, this analysis focused on F and G, F/G, and F + G. Accordingly, the sugar compositions of all eleven authentic honey samples were analyzed using LC–MS, as summarized in Table 1 and Figure S4a,b. The analytical procedure was validated by determining the linear dynamic range and calculating the detection and quantification limits (LOD and LOQ). Linearity was established using a series of standard solutions of glucose and fructose, resulting R^2^ values of 0.991 for glucose and 0.997 for fructose. The repeatability, expressed as relative standard deviation (RSD%), was consistently below 10%, showing acceptable precision. The estimated LOD and LOQ values for glucose are 17.43 and 52.83 mg L^−1^ respectively, while fructose showed LOD and LOQ values of 51.85 and 157.12 mg L^−1^.

In Kuwaiti Sidr honey, F concentrations ranged from 18.18 ± 4.06 to 39.38 ± 2.02 g/100 g, with a mean value of 26.36 ± 6.34 g/100 g. G contents varied between 16.38 ± 4.12 and 33.92 ± 2.18 g/100 g, averaging 24.80 ± 6.35 g/100 g. In addition, across all the authentic honey samples, F/G ranged from 0.97 (S6) to 1.20 (S2), with a mean value of 1.10 and most values being close to or slightly above 1.0.

**Figure 2:**
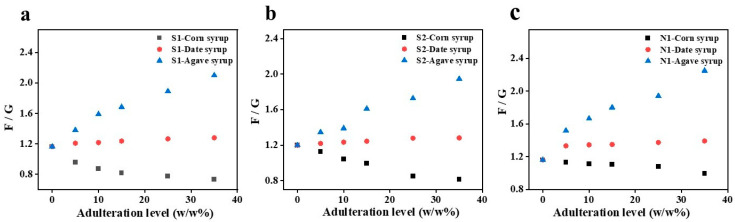
F/G values for Kuwaiti Sidr honey samples (**a**) S1, (**b**) S2, and (**c**) N1 adulterated with corn, date, and agave syrups.

F + G is another essential indicator for honey and is used to ensure compliance with international standards. In honey, F + G can be affected by various factors, including the nectar source, geographical region, bee species, and harvesting techniques used by beekeepers [42]. In this study, F + G varied considerably among the samples, ranging from 34.56 g/100 g (H2) to 73.31 g/100 g (N1), with an average of 50.34 ± 12.03 g/100 g. Notably, authentic reference honeys (S2, S6, and N1) exhibited higher F + G values, with N1 exceeding 70 g/100 g. However, statistical analysis indicated the mean values of F, G, F/G ratio, and total F + G content were not significantly different across all the test honey samples at the 0.05 level (*p* > 0.05). After the preliminary screening of the eleven honey samples (Table 1), three Kuwaiti Sidr honeys (S1, S2, and N1) were selected for adulteration experiments. To evaluate the impacts of the adulterants on the sugar composition and overall quality of the honey and estimate changes in F/G and F + G, each selected authentic honey sample was subjected to controlled adulteration at concentrations ranging from 0% to 35% (*w*/*w*) using corn, agave, and date syrups, as shown in Figure 2a–c and Figure S5a–c, respectively, and detailed in Table 2.

In S1, corn syrup adulteration progressively increased the G concentration from 19.74 ± 1.11 to 24.79 ± 2.02 g/100 g, while decreasing the F concentration from 22.95 ± 0.57 to 18.20 ± 1.54 and F/G from 1.16 to 0.73 (Figure 2a). Despite these changes, F + G remained relatively stable at approximately 43 g/100 g (Figure S5a). In contrast, date syrup adulteration simultaneously increased both F and G concentrations, with F rising from 22.95 ± 0.57 to 27.69 ± 0.62 g/100 g, slightly increasing F/G (1.16–1.28) and increasing F + G (42.69–49.35 g/100 g), as shown in Figure 2a and Figure S5a (slightly increasing both F/G and F + G), respectively. Adulteration with 35% agave syrup markedly increased the F content to 42.27 ± 7.39 g/100 g, while slightly decreasing the G content, substantially altering the sugar profile, doubling F/G to 2.10 (as shown in Figure 2a), and raising F + G to 62.40 g/100 g (as illustrated in Figure S5a).

S2 authentic honey (F/G = 1.20) showed contrasting responses to the different syrups. Adulteration with 35% corn syrup steadily reduced F/G to 0.82, mainly because of the increased G (from 29.91 ± 0.39 to 34.69 ± 0.53 g/100 g) and decreased F (from 35.83 ± 5.75 to 28.30 ± 0.48 g/100 g) contents, as shown in Figure 2b. Date syrup substantially increased both the F and G contents, with F + G increasing from 65.74 to 89.23 g/100 g, while F/G remained between 1.22 and 1.28. However, although agave syrup decreased the G content from 29.91 ± 0.39 to 21.87 ± 0.50 g/100 g, it elevated the F content, markedly increasing F/G to 1.95 (as shown in Figure 2b).

In N1, corn syrup adulteration reduced both the F and G concentrations compared to those in pure honey, decreasing F + G from 73.31 to 52.57 g/100 g (Figure S5c). At higher adulteration levels, F/G approached 1.00, indicating a more balanced monosaccharide profile. In contrast, date syrup adulteration produced the opposite trend: the G contents remained relatively constant, while the F concentrations steadily increased, gradually increasing F/G from 1.16 to 1.39. Again, agave syrup most strongly affected both the F and G contents and F/G, substantially enriching the F content to 48.53 ± 2.25 g/100 g at 35% adulteration and more than doubling F/G to 2.25 (Figure 2c), although F + G remained comparable to that for pure honey (approximately 70–74 g/100 g). These results indicate that adulteration significantly altered the sugar profile of the samples, as reflected by statistically significant differences in F and G contents, F/G ratio, and total sugars (F + G) at *p* < 0.05.

## 4. Discussion

The physicochemical parameters measured in this study (namely, pH, FA, EC and moisture content) are widely recognized indicators of honey quality and authenticity. These parameters represent the organic acid content and ionic strength of honey, both of which are highly sensitive to dilution or substitution with plant syrups. Such adulteration practices can substantially alter physicochemical properties (including pH, FA, and EC) and change the concentrations of components such as sugars, HMF, and ash [43,44]. Therefore, the interpretation and comparison of these results against Codex Alimentarius and IHC criteria can provide a robust framework for detecting adulteration and ensuring product integrity [43,45,46].

Honey’s acidity is primarily derived from various organic acids, including gluconic, tartaric, malic, citric, and succinic acids, which also contribute to honey’s antimicrobial activity by inhibiting microbial growth. Additionally, the pH of honey can be influenced by both extraction methods and storage conditions, which, in turn, affect honey’s stability and shelf life. EC, which measures the ability of a solution to carry an electric current, is closely linked to honey’s ion concentration, is determined by honey’s botanical origin and mineral and inorganic ion contents [47], and is affected by other components, such as moisture, water-insoluble solids, organic acids, proteins, sugars, polyols, and pollen grains, all of which can contribute to electrolytes and impact the overall conductivity [48]. Moreover, the moisture content of honey sample influences its stability and shelf life. Lower moisture levels enhance preservation, while higher levels increase the risk of fermentation to ethanol during storage [41].

Most authentic honey samples exhibited pH values within/or very close to the World Health Organization’s (WHO’s) recommended range of 3.4–6.1 [49], except for S1 (6.49 ± 0.21) and N3 (6.29 ± 0.06), which slightly exceeded this upper limit. These findings are consistent with those in previous reports on Sidr honey [40]. FA values of all Kuwaiti authentic honey samples varied from 6.75 ± 0.21 to 23.61 ± 0.00 mEq kg^−1^ (mean 16.80 ± 4.57 mEq kg^−1^) which were within the limit (below 50 mEq kg^−1^) according to the WHO [40]. FA values previously reported for Sidr honey showed considerable variability. Some studies have documented higher FA values, such as 36.50 ± 0.05 and 37.50 ± 0.05 mEq kg^−1^ for Egyptian and Saudi honeys, respectively [50]. Although the European Commission recommends a maximum EC of 800 µS cm^−1^, Sidr honey is known to exhibit higher conductivities than other monofloral honeys. Thus, the elevated EC values of the authentic Kuwaiti honeys in this study are characteristic of Sidr honey [40]. Importantly, all honey samples were collected during the single Sidr flowering season in Kuwait (November 2023–February 2024) from regions where *Ziziphus spina-christi* represents the predominant nectar source, and Sidr honey is the primary monofloral product harvested during this period. Variations observed in physicochemical parameters, particularly pH and electrical conductivity (EC), including the lower values recorded for samples S1 and S5, do not necessarily indicate a different floral origin, as both parameters are known to exhibit wide natural variability in Sidr honeys depending on environmental factors such as soil composition, salinity, climate, moisture level, and hive management practices. Notably, the pH (5.12–6.49) and EC (429–1121 μS/cm) values obtained in this study fall within ranges previously reported for Sidr honey. The moisture content of all Kuwaiti honey samples also remained within the acceptable limit of ≤20% recommended by Codex Alimentarius [49]. These findings are consistent with the moisture content reported for Sidr honey samples from different origins, as presented by Roshan et al. [40]. Furthermore, one sample *t*-tests showed no statistically significant difference in pH, FA, EC and moisture content from their respective overall mean values (*p* > 0.05), indicating consistency among the examined Kuwaiti honey samples.

### 4.1. Evaluation of the Physicochemical Properties of Adulterated Honeys

After the honeys were adulterated with the syrups, the pH values of the adulterated honeys remained relatively stable, only slightly varying. In particular, corn syrup slightly increased the pH values of S2 and N1, as shown in Figure 1a and Figure S3a,b, respectively, consistent with trends reported in previous studies [10,51], while the pH of S1 did not measurably change. In contrast, although date syrup slightly decreased the pH of S1, it modestly increased the pH values of S2 and N2. Agave syrup minimally impacted the honeys’ pH values, as the pH of none of the Kuwaiti Sidr honeys noticeably changed, similar to findings reported by Ciursa et al. [52]. Overall, none of the syrups substantially shifted the pH. This stability may be attributed to the buffering capacity of the honey matrix, which resisted marked changes in acidity and maintained the pH values of the adulterated samples within a relatively narrow range [10]. Some previous studies have found that at higher adulteration levels, sugar syrups, particularly G–F or high-F corn syrups, increased the pH [10,53]. However, acidic adulterants, such as hydrolyzed inulin or invert syrups, may, instead, slightly decrease the pH [52,54]. Collectively, these results showed that in adulterated honey, pH changes are governed by the chemical properties and buffering interactions of both the honey and syrup.

In honey, FA primarily arises from organic acids, which, although only account for approximately 0.5% of the total composition, play important roles in defining the flavor, stability, and chemical properties of honey [55]. FA is a widely recognized indicator of fermentation, as improper storage, excessive heating, microbial contamination, or the breakdown of sugars by osmophilic yeasts may elevate FA levels. In this study, the FAs of the authentic S1, S2, and N1 honeys were 6.75 ± 0.53, 20.67 ± 2.20, and 20.24 ± 2.65 mEq kg^−1^, respectively. FA was negatively correlated with the volumes of corn and agave syrups added and positively correlated with the volume of date syrup added, as shown in Figure 1b and Figure S3c,d, respectively. Overall, across all the adulterated honeys, FA ranged from 4.50 ± 1.06 to 32.69 ± 2.00 mEq kg^−1^, depending on the adulterant type and proportion. Similar trends were reported by Oroian et al. [56], who showed that 5–50% G, hydrolyzed inulin syrup, malt wort, and invert syrup increased the FA of honey, while F negligibly affected it. In their study, FA rose from 19.44 to 162.88 mEq kg^−1^ for pure honey and at 50% adulteration, respectively. Additionally, Gün and Karaoğlu reported negative correlations between the FA of blossom honey and the volumes of G–F/cornstarch (GFCS) and maltose/cornstarch (MCS) syrups added [10]. According to the Turkish Food Codex Communiqué on Honey [57] and Council Directive (2001) [58] honey should have an FA value of below 50 mEq kg^−1^, with no specified minimum limit. In this study, all the authentic and adulterated honeys remained below this regulatory threshold, indicating that no adulteration was detected in any of the samples.

EC is a physicochemical parameter that indicates honey’s mineral content and varies directly with the contents of mineral substances, organic acids, and other organic compounds [59]. Adulterants such as date, corn, and agave syrups can substantially influence EC, either elevating or reducing it, depending on the mineral composition, as shown in Figure 1c and Figure S3e,f, respectively. Therefore, because the authentic honeys in this study were rich in minerals, such as potassium, iron, magnesium, and calcium, date syrup substantially elevated EC [60]. In contrast, corn and agave syrups slightly lowered EC [61]. Similarly, Ciursa et al. [52] found that agave and corn syrups reduced the EC of raspberry honey. Another study reported that for blossom honey, the EC and both the GFCS and MCS concentrations were strongly negatively correlated [10], whereas a separate study found that the ECs of various honeys, including acacia, Tilia, and polyfloral, were influenced by the adulterant type: F syrup reduced EC, while hydrolyzed inulin syrup increased EC with increasing concentration [56]. Although the European Commission recommends a maximum EC limit of 800 µS cm^−1^ for honey, several studies have reported that Sidr honey often exceeds this threshold, which is a typical characteristic of this floral type [40,50,62].

Additionally, the effect of adulteration on moisture was evaluated, as shown in Figure 1d and Figure S3g,h. Pure syrups exhibited higher moisture levels than honey, which explains the observed increase in moisture when these syrups were added. Corn syrup adulteration resulted in relatively stable moisture levels across all honey samples, whereas date and agave syrups caused slight increase with higher adulteration levels. The moisture content observed in the adulterated Kuwaiti honey samples agrees with previous findings reported for honey adulteration studies [63,64].

These findings indicate that physicochemical parameters, such as pH, FA, EC and moisture content, responded differently to various adulterants in Kuwaiti Sidr honey: Agave syrup negligibly affected both pH and FA while reducing EC, and it caused a slight increase in moisture content at higher adulteration levels. Corn syrup slightly changed the pH and consistently reduced FA and maintained relatively stable moisture levels across samples. In contrast, date syrup notably raised FA, EC and moisture content, reflecting its distinct mineral and organic acid composition. To quantitatively confirm these observations, the impact of adulteration level on physicochemical parameters was assessed using one-way ANOVA, with effect sizes reported as η^2^ (Table S3). EC showed the strongest and most consistent response across all syrup types, with very large effects observed for corn (F = 852.48, *p* < 0.0001, η^2^ = 0.9922), date (F = 22.61, *p* < 0.0001, η^2^ = 0.7723), and agave (F = 147.88, *p* < 0.0001, η^2^ = 0.9568). These results indicate that 77–99% of the variance in EC was explained by adulteration level, confirming EC as the most sensitive physicochemical indicator of syrup addition within the tested concentration range. In contrast, FA exhibited a statistically significant response only to corn syrup adulteration (F = 3.52, *p* = 0.0339, η^2^ = 0.3455) across all the tested honey samples, while adulteration with date (F = 3.04, *p* = 0.0529, η^2^ = 0.3130) and agave (F = 2.16, *p* = 0.1241, η^2^ = 0.2450) syrups were not result in significant changes in FA. Moreover, pH and moisture content did not show statistically significant differences across adulteration levels for any syrup type (*p* > 0.05). For pH, although moderate effect size values were observed (η^2^ ~ 0.31), the measured changes remained within a relatively narrow range, indicating limited sensitivity to syrup addition under the studied conditions. In contrast, moisture content exhibited small effect sizes (corn, η^2^ = 0.0953; date, η^2^ = 0.0246; agave, η^2^ = 0.0108) and consistently high *p*-values, confirming minimal variation with increasing adulteration. These findings suggest that, within the tested concentration range, EC is very sensitive parameter for detecting adulteration in honey samples.

In addition to agave, date, and corn syrups, other common adulterants, such as rice, maple, and inverted sugar syrups, can substantially alter the physicochemical properties of honey. According to Ciursa et al. [52], when honey was adulterated with rice, maple and inverted sugar syrups, the pH remained relatively stable, negligibly changing across different syrup concentrations. However, rice syrup notably increased FA, indicating a higher contribution of organic acids at elevated adulteration levels. EC substantially rose in honey adulterated with maple and rice syrups, likely because of the honey’s higher mineral or ionic content. In contrast, EC progressively decreased in honey adulterated with agave, corn, and inverted sugar syrups, indicating the dilution of the honey’s natural mineral composition. Our experimental findings were consistent with those in previous reports, emphasizing that physicochemical parameters respond differently depending on the chemical properties of the adulterant. Traditional physicochemical indicators were developed to evaluate honey quality and freshness rather than to detect intentional adulteration, and modern syrups are increasingly formulated to mimic the natural properties of genuine honey. As a result, standard quality parameters alone may lack the sensitivity and specificity required to identify complex fraud [20,65].

Overall, our findings emphasize the necessity of a multi-parameter approach to authenticity assessment. While routine physicochemical tests remain useful for quality control, they should be complemented by more discriminating indicators, such as sugar profiling (F, G, F/G ratio, and total F + G). Integrating these approaches increases the reliability of detecting modern honey adulteration practices, which continue to evolve in complexity.

### 4.2. LC–MS Evaluation of Fructose and Glucose Contents

Carbohydrates are the primary constituents of honey, accounting for approximately 95% of honey’s dry weight, with monosaccharides (mainly F and G) representing approximately 75% and the remainder comprising various disaccharides (e.g., sucrose) and oligosaccharides up to tetrasaccharides [66]. Advanced chromatographic methods, particularly GC–MS, have been widely used to characterize the sugar profiles of honeys and even detect traces of complex carbohydrates. Using GC–MS, Ruiz-Matute et al. [67] identified 12 trisaccharides in Spanish honey, including planteose and α-3′-glucosyl-isomaltose, both reported for the first time. Additionally, the authors detected 10 tetrasaccharides, notably, nystose and six sucrose-derived compounds.

Although previous studies have shown that F and G were the most abundant sugars in all the samples [20,28], F/G is widely used to evaluate both honey quality and authenticity. For honey, although the F/G range is usually between 1 and 1.2, it varies depending on the nectar source from which the honey was extracted [68]. For example, Sidr honey typically shows F/G values ranging from 0.9 to 1.35 [41,69], those of blossom honey range from 0.9 to 1.4 [57], and those of Israeli honey fall between 1.11 and 1.38 [70]. Values outside these ranges potentially indicate either adulteration or uncommon nectar sources. In our study, because all the samples fell within the typical Sidr honey F/G range, the data were consistent with authentic Sidr honey. Additionally, F + G is important for evaluating honey quality, as high F and G contents are characteristic of genuine honey and contribute to its sweetness, viscosity, and shelf stability. According to Codex Alimentarius, floral honey has a minimum F + G requirement of 60 g/100 g [46]. As deviations from this standard may indicate either adulteration or natural flower-origin-related variations, F + G is an essential metric in honey authentication [42]. Table 3 compares the sugar contents of Sidr honeys collected from different geographical regions. The honeys’ F and G contents ranged from 26.36 and 24.30 to 39.70 and 31.50 g/100 g, respectively, and their corresponding F/G values ranged between 1.10 and 1.42, respectively, indicating considerable variation among the Sidr honeys. Although most reported Sidr honeys exhibit total reducing sugars F + G values above 58 g/100 g, the Kuwaiti Sidr honeys analyzed in this study showed comparatively lower contents. This variability likely reflects the combined influence of Kuwait’s arid climate and high temperatures, limited nectar availability, and post-harvest handling practices on sugar composition [30]. Such regional effects are consistent with published Sidr honey data from other geographic locations and highlight the importance of detailed sugar profiling in evaluating honey quality and provenance. Although some samples fell below the CODEX minimum requirement of 60% for F + G, their F/G ratios (0.97–1.20) remained within the characteristic range reported for authentic Sidr honey. Collectively, these findings indicate that the observed compositional variability reflects natural heterogeneity associated with environmental and regional factors rather than differences in floral origin. Moreover, one-sample statistical analysis confirmed that, at the 0.05 significance level, the population means of F, G, F/G ratio, and total F + G content were not significantly different from the corresponding test means (*p* > 0.05), further supporting their authenticity.

According to the results of the initial screening of the eleven Sidr honeys, as shown in Table 1, S1, S2 and N1 were selected as representative samples for adulteration experiments spanning the observed F + G range. Because S1 had the lowest F + G value (42.69 g/100 g; F/G = 1.16), it was the representative lower-sugar model. S2 had an intermediate F + G value (65.74 g/100 g; F/G = 1.20), representative of mid-range compositions. N1 had the highest F + G value (>73 g/100 g; F/G = 1.16), slightly above the Codex Alimentarius Commission’s minimum threshold for reducing sugars in blossom honeys [39]. These samples provided distinct baseline sugar compositions against which to evaluate the effects of different syrup adulterants, as listed in Table 2.

The adulteration experiments revealed apparent and consistent syrup-type-dependent trends in the sugar composition, highlighting the effectiveness of sugar profiling as a reliable approach for honey authentication. In our study, across all the authentic Kuwaiti Sidr honeys, corn syrup adulteration consistently lowered F/G, consistent with both high G (18.13 g/100 g) and negligibly changing F contents [29], as shown in Figure 2a–c. In contrast, date syrup adulteration increased both F and G contents, modestly increasing F/G (Figure 2a–c) and indicating that date syrup adulteration may be more challenging to detect solely based on F/G, as the values remained within the accepted range for authentic Sidr honeys. However, date syrup notably increased F + G.

This effect was even more pronounced for agave syrup, where, at 35% adulteration, F/G ≥ 2.0 because agave syrup was rich in F (40.95 g/100 g) and contained negligible G (Figure 2a–c). Correspondingly, although the absolute F concentration slightly decreased for corn syrup adulteration, it substantially increased for agave syrup adulteration. These findings aligned with those by Oroian et al. [9], who reported that the agave syrup adulteration of Tilia honey increased F/G, while rice syrup adulteration decreased it. Similarly, corn and maple syrups substantially reduced both F and G contents, indicating that the impact of adulteration on the honey composition strongly depended on both the adulterant type and sugar profile. Moreover, one-way ANOVA confirmed that sugar composition was highly sensitive to adulteration level (Table S3). For corn syrup, F, G, F/G ratio, and total F + G content showed statistically significant changes (*p* < 0.05), with large effect sizes (η^2^ = 0.51–0.88), indicating a strong influence of adulteration level on sugar composition. Similarly, date syrup significantly affected F, G, F/G ratio, and F + G (*p* < 0.05), with large-to-very large effect sizes (η^2^ = 0.50–0.91). Among these, total F + G content consistently showed the strongest response. Agave syrup also significantly influenced F, G, and F + G (*p* < 0.05), with large effect sizes (η^2^ = 0.37–0.78). However, the F/G ratio was not significantly affected by agave syrup adulteration (*p* = 0.6296, η^2^ = 0.0598), indicating minimal change in this parameter across the tested levels. These variations revealed that corn, date, and agave syrups not only influence physicochemical properties of honey but also induce detectable changes in its sugar profile.

## 5. Conclusions

This study evaluated the physicochemical characteristics and sugar composition of authentic and adulterated Kuwaiti Sidr honeys using standard quality parameters and LC–MS analysis. All authentic honey samples exhibited acceptable moisture contents and physicochemical values within or close to established quality ranges, with no statistically significant differences in pH, FA, EC, or moisture content from their respective overall mean values (*p* > 0.05).

Adulteration experiments revealed that syrup addition alters the physicochemical and sugar composition of Sidr honey, with each adulterant producing distinct effects. Among the evaluated parameters, EC emerged as the most sensitive physicochemical indicator, showing statistically significant differences across all adulteration levels for corn, date, and agave syrups (*p* < 0.05). FA showed significant changes only with corn syrup adulteration, whereas pH and moisture content did not show any statistically significant difference across adulteration levels for all syrup types (*p* > 0.05). Sugar profiling using LC–MS proved more effective for identifying adulteration, with significant differences (*p* < 0.05) detected in F, G, the F/G ratio, and F + G content across adulterated samples. Although some authentic Kuwaiti sidr honeys exhibited slightly lower F + G values than the typical Codex thresholds, their F/G ratios together with the absence of any statistically significant differences from the overall population means, supported their authenticity and suggested that the observed variability was due to natural regional factors. In adul-terated samples, corn syrup consistently reduced the F/G ratio, whereas date syrup in-creased the total F + G content. This effect was the most pronounced for agave syrup, which, because of its high F (~41 g/100 g) and negligible G contents, elevated the F/G ratio to ≥2.0 at 35% adulteration.

Overall, although adulteration can affect several physicochemical properties, our findings suggested that sugar composition analysis provides deeper and more discriminative insights into adulteration practices. Additionally, a multiparameter analytical framework that combines physicochemical indices with targeted sugar profiling can enhance adulteration detection accuracy, support robust honey authentication, and strengthen consumer protection and market integrity.

Future work should expand the sample size and include a broader range of adulterants to enable the application of multivariate chemometric approaches, such as principal component analysis (PCA), to identify variables associated with honey adulteration and enhance the discrimination between authentic and adulterated samples. Additionally, integrating insights from recent LC-MS-based authenticity studies, including LC-HRMS and metabolomics-driven approaches, will further strengthen analytical strategies and improve the robustness of future authenticity evaluations.

## Figures and Tables

**Table 1:** F and G (g/100 g), F/G, and F + G (g/100 g) for various Kuwaiti Sidr honey samples analyzed using LC–MS. Values are presented as means ± standard deviations (SDs).

Sample	F (g/100 g)	G (g/100 g)	F/G	F + G
S1	22.95 ± 0.57	19.74 ± 1.11	1.16	42.69
S2	35.83 ± 5.75	29.91 ± 0.39	1.20	65.74
S3	23.09 ± 2.80	20.12 ± 3.62	1.15	41.74
S4	25.07 ± 4.13	32.90 ± 7.69	0.99	50.41
S5	26.17 ± 2.40	26.26 ± 2.67	1.00	52.43
S6	31.96 ± 7.09	32.90 ± 7.69	0.97	64.86
N1	39.38 ± 2.02	33.92 ± 2.18	1.16	73.31
N2	24.69 ± 2.04	23.83 ± 2.38	1.04	48.52
N3	21.92 ± 1.98	19.02 ± 1.86	1.15	40.93
H1	20.76 ± 4.80	17.82 ± 4.00	1.16	38.59
H2	18.18 ± 4.06	16.38 ± 4.12	1.11	34.56
Mean	26.36 ± 6.34	24.80 ± 6.35	1.10	50.34 ± 12.03
Minimum	18.18 ± 4.06 (H2)	16.38 ± 4.12 (H2)	0.99 (S4)	34.56 (H2)
Maximum	39.38 ± 2.02 (N1)	33.92 ± 2.18 (N1)	1.20 (S2)	73.31 (N1)

**Table 2:** F and G (g/100 g), F + G (g/100 g), and F/G (measured using LC–MS) for Kuwaiti Sidr honeys adulterated with corn, date, or agave syrup. Values are expressed as means ± SDs (*n* = 6).

**S1**					
**Adulterant**	**Adulteration Level (%)**	**F (g/100 g)**	**G (g/100 g)**	**F/G**	**F + G (g/100 g)**
	0	22.95 ± 0.57	19.74 ± 1.11	1.16	42.69
Corn	5	21.21 ± 2.54	22.21 ± 2.14	0.96	43.43
	10	20.34 ± 6.06	23.33 ± 2.06	0.87	43.67
	15	19.30 ± 4.77	23.65 ± 2.66	0.82	42.95
	25	18.89 ± 2.92	24.30 ± 0.99	0.78	43.19
	35	18.20 ± 1.54	24.79 ± 2.02	0.73	42.99
Date	5	23.86 ± 1.42	19.75 ± 3.39	1.21	43.61
	10	25.45 ± 1.36	20.92 ± 0.89	1.22	46.37
	15	26.95 ± 4.53	21.80 ± 4.01	1.24	48.75
	25	26.97 ± 2.05	21.32 ± 3.12	1.27	48.29
	35	27.69 ± 0.62	21.66 ± 1.91	1.28	49.35
Agave	5	26.54 ± 3.59	19.22 ± 3.38	1.38	45.77
	10	29.96 ± 0.83	18.85 ± 0.45	1.59	48.81
	15	32.92 ± 5.60	19.57 ± 2.06	1.68	52.49
	25	38.85 ± 0.57	20.55 ± 2.50	1.89	59.40
	35	42.27 ± 7.39	20.13 ± 1.79	2.10	62.40
**S2**					
**Adulterant**	**Adulteration Level (%)**	**F (g/100 g)**	**G (g/100 g)**	**F/G**	**F + G**
	0	35.83 ± 5.75	29.91 ± 0.39	1.20	65.74
Corn	5	34.81 ± 3.44	30.97 ± 3.29	1.12	65.78
	10	33.47 ± 1.43	32.12 ± 5.13	1.04	65.59
	15	33.01 ± 1.16	33.21 ± 2.15	0.99	66.21
	25	29.21 ± 3.13	34.40 ± 0.25	0.85	63.61
	35	28.30 ± 0.48	34.69 ± 0.53	0.82	62.99
Date	5	41.28 ± 2.74	33.87 ± 1.46	1.22	75.15
	10	43.92 ± 5.22	35.61 ± 1.26	1.23	79.53
	15	45.75 ± 0.91	36.78 ± 6.60	1.24	82.51
	25	46.38 ± 8.78	36.30 ± 0.42	1.28	82.68
	35	50.11 ± 7.86	39.12 ± 4.27	1.28	89.23
Agave	5	38.55 ±7.3	28.66 ± 9.57	1.34	67.21
	10	33.91 ± 0.74	24.39 ± 4.44	1.39	58.30
	15	38.00 ± 0.97	23.60 ± 1.60	1.61	61.60
	25	40.09 ± 3.68	23.23 ± 5.67	1.73	63.31
	35	42.53 ± 2.34	21.87 ± 0.50	1.95	64.40
**N1**					
**Adulterant**	**Adulteration Level (%)**	**F (g/100 g)**	**G (g/100 g)**	**F/G**	**F + G**
	0	39.38 ± 2.02	33.92 ± 2.18	1.16	73.31
Corn	5	31.24 ± 2.91	27.62 ± 1.72	1.13	58.86
	10	29.36 ± 3.44	26.37 ± 0.77	1.11	55.73
	15	28.89 ± 1.95	26.18 ± 1.37	1.10	55.07
	25	27.79 ± 0.49	25.71 ± 2.18	1.08	53.50
	35	26.24 ± 1.53	26.33 ± 4.29	1.00	52.57
Date	5	36.46 ± 0.99	27.35 ± 1.52	1.33	63.81
	10	36.40 ± 0.63	27.07 ± 3.29	1.34	63.47
	15	36.44 ± 5.07	27.01 ± 0.71	1.35	63.46
	25	36.55 ± 5.43	26.60 ± 1.24	1.37	63.16
	35	36.38 ± 2.71	26.12 ± 2.43	1.39	62.50
Agave	5	44.71 ± 2.56	29.44 ± 3.96	1.52	74.15
	10	45.21 ± 4.75	27.16 ± 1.23	1.67	72.37
	15	46.73 ± 1.23	25.95 ± 1.79	1.80	72.69
	25	48.18 ± 1.94	24.82 ± 0.94	1.94	73.00
	35	48.53 ± 2.25	21.57 ± 1.45	2.25	70.10

**Table 3:** Sugar profiles of Sidr honeys collected from different geographical regions, as reported in previous studies [41,62,71].

Geographical Region	F (g/100 g)	G (g/100 g)	F/G	F + G (g/100 g)
United Arab Emirates	33.26 ± 0.48	25.06 ± 1.15	1.33	58.33 ± 1.28
China	35.02 ± 0.62	26.64 ± 0.70	1.31	61.66 ± 1.16
Iraq	36.01 ± 1.05	28.89 ± 0.11	1.25	64.90 ± 0.95
Pakistan	34.43 ± 0.48	25.70 ± 0.83	1.26	60.14 ± 1.10
Bashawer	33.94 ± 1.13	27.67 ± 0.78	1.22	61.61 ± 1.61
Panjab	33.48 ± 0.46	25.61 ± 0.96	1.31	59.10 ± 1.18
Saudi Arabia	34.94 ± 0.31	27.53 ± 0.27	1.27	62.47 ± 0.52
Saudi Arabia	39.70 ± 0.6	31.50 ± 0.70	1.27	71.20 ± 0
Kashmir	33.38 ± 1.21	25.77 ± 0.89	1.29	59.16 ± 1.93
Libya	35.99 ± 0.66	25.61 ± 1.14	1.4	61.61 ± 1.64
Egypt	33.69 ± 0.91	26.02 ± 0.98	1.29	59.71 ± 1.61
Upper Egypt (Luxor, Qena, and Sohag)	34.38 ± 3.12	24.30 ± 2.60	1.42	58.68 ± 2.01
India	32.79 ± 0.64	24.77 ± 0.65	1.32	57.56 ± 0.91
Yemen	34.73 ± 0.52	27.99 ± 0.48	1.24	62.72 ± 0.83
Kuwait (This study)	26.36 ± 6.34	24.80 ± 6.35	1.10	50.34 ± 12.03

## Data Availability

The original contributions presented in the study are included in the article/Supplementary Materials, further inquiries can be directed to the corresponding authors.

## Supplementary Materials

The following supporting information can be downloaded at: https://www.mdpi.com/article/10.3390/foods15061038/s1, Figure S1: Calibration curve for (**a**) fructose and (**b**) glucose standards analyzed using LC-MS; Figure S2: Average values of (**a**) pH (**b**) free acidity (**c**) electrical conductivity and (**d**) moisture content of Kuwaiti Sidr honey samples; Figure S3: Evaluation of physicochemical parameters in S2 and N1 Kuwaiti Sidr honey samples adulterated with corn, date, and agave syrups: (**a**,**b**) pH, (**c**,**d**) free acidity, (**e**–**f**) electrical conductivity and (**g**–**h**) moisture content. Error bars represent one standard deviation; Figure S4: (**a**) F/G ratio and (**b**) F + G (g/100 g) for various Kuwaiti Sidr honey samples analyzed using LC–MS; Figure S5: Total sugar content of three different Kuwaiti sider honey samples (**a**) S1 (**b**) S2 and (**c**) N1 adulterated with corn, date and agave syrups. Table S1: Physiochemical parameters of unadulterated Kuwaiti Sidr honey: pH, FA, EC and moisture content; Table S2: The physicochemical analysis of Kuwaiti honey samples adulterated with corn, date and agave syrups; Table S3: One-way ANOVA results for the effects of corn, date, and agave syrup adulteration on honey physicochemical parameters and sugar composition. Reported statistics include F-values, exact p-values, and effect sizes (η^2^).
