# Supplementary material for: Evaluating Honey Adulteration Through Physicochemical Characterization and Liquid Chromatography–Mass Spectrometry-Based Sugar Profiling
Source: Foods. 2026 Mar 16;15(6):1038. doi: 10.3390/foods15061038 (PMC13024881; doi:10.3390/foods15061038)
Supplement: Supplementary file 1 [file foods-15-01038-s001.zip › foods-4167668-supplementary.pdf]

Supporting information

Evaluating Honey Adulteration Through Physicochemical Characterization and Liquid Chromatography-Mass Spectrometry-Based Sugar Profiling

Entesar Al-Hetlani[a]*, Bessy D`Cruz[a], Mohammed Hayssam[a], Bedraya Mandekar[b], Mohamed O. Amin[c]*

[a] Department of Chemistry, College of Science, Kuwait University, Sabah Al Salem University City P.O. Box 5969, Safat 13060, Shadadiya, Kuwait

[b] Environmental & Life Science research Center, Kuwait Institute for Scientific Research, Kuwait

[c] Department of Chemistry, University at Albany, State University of New York, Albany, NY, USA

Corresponding authors:

Entesar Al-Hetlani: entesar.alhetlani@ku.edu.kw

Mohamed O. Amin: moamin@albany.edu

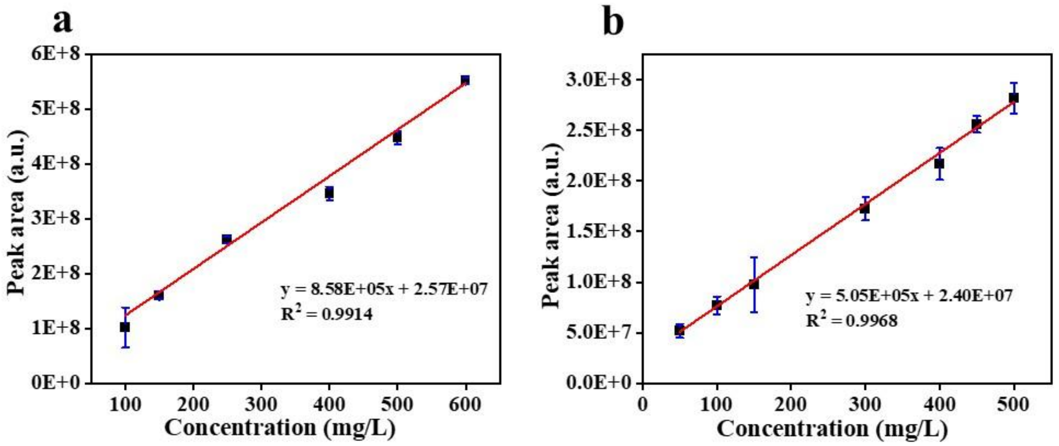

Figure S1. Calibration curve for (**a**) fructose and (**b**) glucose standards analyzed using LC-MS.

Table S1: Physiochemical parameters of unadulterated Kuwaiti Sidr honey: pH, FA, EC and moisture content.

Sample	pH	Free acidity (mEq Kg^{-1})	Conductivity (µs cm^{-1})	Moisture content (%)
**S1**	6.49 ± 0.21	6.75 ± 0.21	1121.5 ± 30.41	19.77 ± 1.91
**S2**	5.06 ± 0.01	20.67 ± 2.20	460.60 ± 5.09	15.88 ± 0.38
**S3**	6.03 ± 0.06	15.55 ± 0.80	1169.00 ± 125.87	16.11 ± 0.65
**S4**	5.80 ± 0.03	19.4 ± 0.13	1378.50 ± 0.71	14.14 ± 0.09
**S5**	5.12 ± 0.00	18.18 ± 0.53	429.15 ± 13.93	18.86 ± 0.55
**S6**	5.70 ± 0.01	23.61 ± 0.00	1360.50 ± 3.54	16.60 ± 0.20
**N1**	5.62 ± 0.11	20.24 ± 2.65	981.30 ± 44.83	13.03 ± 1.13
**N2**	5.94 ± 0.04	16.87 ± 1.06	1299.00 ± 29.70	16.16 ± 0.55
**N3**	6.29 ± 0.06	12.26 ± 0.16	1385.50 ± 139.30	14.14 ± 0.75
**H1**	5.81 ± 0.01	16.3 ± 0.79	1308.50 ± 44.55	17.50 ± 1.19
**H2**	5.62 ± 0.05	14.99 ± 1.59	834.80 ± 108.05	15.93 ± 0.37
**Mean**	5.77 ± 0.43	16.80 ± 4.57	1066.21 ± 336.56	15.60 ± 0.67
**Minimum**	5.06 ± 0.01	6.75 ± 0.21	429.15 ± 13.93	13.03 ± 1.13
**Maximum**	6.49 ± 0.21	23.61 ± 0.00	1385.50 ± 139.30	19.77 ± 1.91

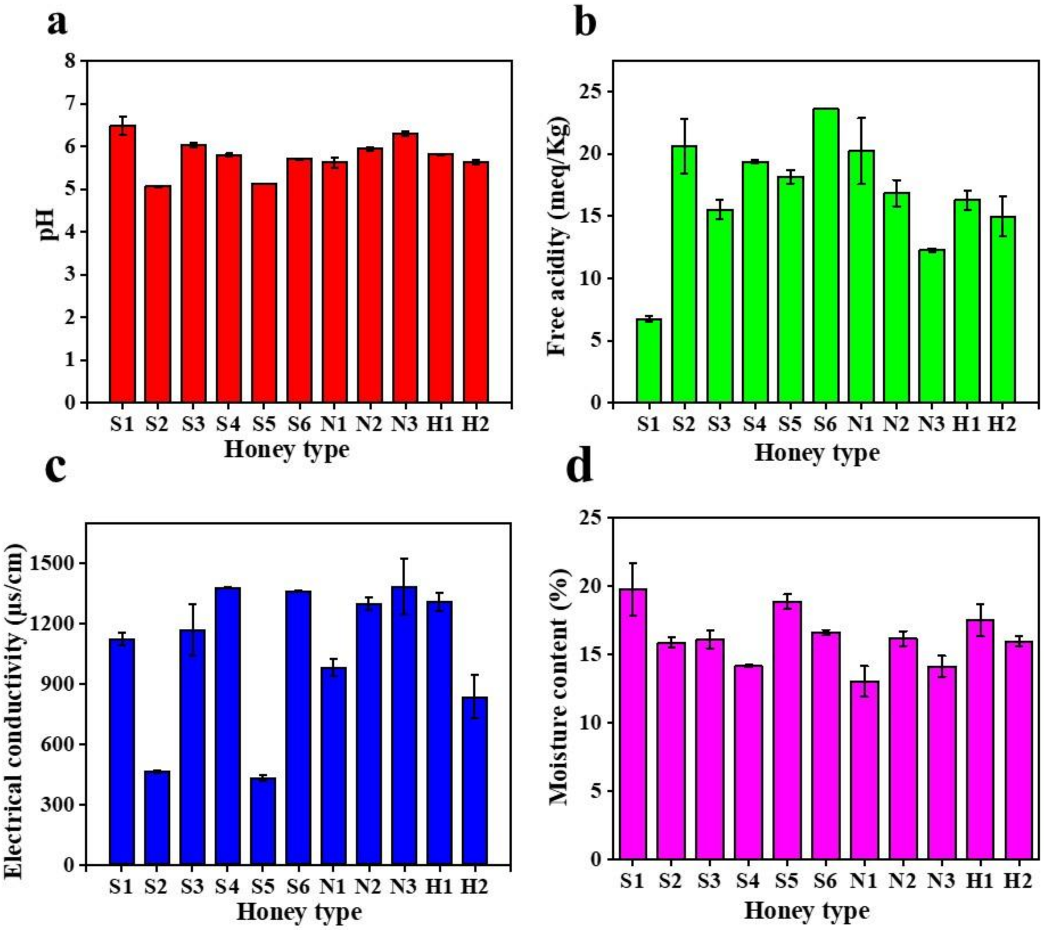

Figure S2. Average values of (a) pH (b) free acidity (c) electrical conductivity and (d) moisture content of Kuwaiti Sidr honey samples.

Table S2. The physicochemical analysis of Kuwaiti honey samples adulterated with corn, date and agave syrups.

S1					
**Adulterants**	**Adulteration level (%)**	**pH**	**Free acidity (mEq Kg^{-1})**	**Conductivity (μs cm^{-1})**	**Moisture content (%)**
**Corn syrup**	0	6.49 ± 0.21	6.75 ± 0.53	1121.50 ± 30.41	19.77 ± 1.91
	5	6.73 ± 0.28	5.06 ± 1.33	1113.50 ± 37.48	18.50 ± 0.01
	10	6.75 ± 0.21	4.96 ± 0.93	1100.00 ± 31.11	18.38 ± 0.79
	15	6.69 ± 0.18	4.87 ± 1.06	1097.00 ± 28.28	18.34 ± 1.44
	25	6.65 ± 0.18	4.86 ± 1.07	1093.50 ± 27.58	18.41 ± 0.87
	35	6.59 ± 0.15	4.50 ± 1.06	1090.00 ± 26.87	17.48 ± 1.11
**Date syrup**	5	6.45 ± 0.12	10.78 ± 2.52	1287.00 ± 41.01	15.27 ± 0.71
	10	6.33 ± 0.12	12.93 ± 2.39	1438.00 ± 21.21	15.65 ± 1.36
	15	6.27 ± 0.13	15.09 ± 1.72	1579.00 ± 62.23	16.03 ± 0.96
	25	6.23 ± 0.13	19.86 ± 2.12	1886.00 ± 32.53	16.07 ± 1.47
	35	6.17 ± 0.09	25.12 ± 1.06	2119.50 ± 122.33	16.55 ± 1.56
**Agave syrup**	5	6.49 ± 0.28	6.37 ± 0.53	1055.00 ± 38.18	14.47 ± 1.53
	10	6.47 ± 0.19	6.19 ± 0.27	1005.90 ± 24.18	14.33 ± 0.52
	15	6.46 ± 0.18	6.09 ± 0.13	965.05 ± 25.10	14.44 ± 0.47
	25	6.43 ± 0.17	6.00 ± 0.53	861.25 ± 19.73	15.27 ± 0.82
	35	6.43 ± 0.20	5.81 ± 0.80	774.35 ± 34.58	15.17 ± 0.74
**S2**					
**Adulterants**	**Adulteration level (%)**	**pH**	**Free acidity (mEq Kg^{-1})**	**Conductivity (μs cm^{-1})**	**Moisture content (%)**
**Corn syrup**	0	5.06 ± 0.01	20.67 ± 2.20	464.55 ± 0.49	15.88 ± 0.38
	5	5.19 ± 0.12	14.61 ± 3.60	492.30 ± 3.39	14.46 ± 0.71
	10	5.20 ± 0.14	13.94 ± 3.71	520.45 ± 3.04	14.21 ± 0.13
	15	5.21 ± 0.13	13.77 ± 3.47	560.45 ± 1.20	14.31 ± 0.29
	25	5.27 ± 0.13	12.72 ± 3.61	609.10 ± 5.09	14.58 ± 0.10
	35	5.34 ± 0.11	11.87 ± 2.66	679.10 ± 0.85	14.96 ± 0.40
**Date syrup**	5	5.55 ± 0.01	22.24 ± 0.54	687.35 ± 28.35	12.47 ± 3.58
	10	5.65 ± 0.06	23.57 ± 0.26	866.70 ± 32.53	14.16 ± 3.40

	15	5.69 ± 0.05	24.12 ± 0.53	1071.50 ± 0.71	14.77 ± 0.13
	25	5.78 ± 0.02	25.25 ± 0.52	1441.50 ± 10.61	15.35 ± 0.01
	35	5.84 ± 0.00	28.64 ± 0.00	1760.00 ± 50.91	15.79 ± 0.47
**Agave syrup**	5	5.32 ± 0.01	14.20 ± 1.63	434.95 ± 3.32	14.2 ± 0.06
	10	5.37 ± 0.04	13.58 ± 1.19	417.35 ± 4.17	14.29 ± 0.14
	15	5.34 ± 0.01	11.76 ± 0.74	399.70 ± 1.13	14.76 ± 0.66
	25	5.34 ± 0.04	10.62 ± 0.26	359.45 ± 1.34	15.21 ± 0.01
	35	5.33 ± 0.00	9.94 ± 0.86	325.10 ± 1.56	16.70 ± 0.17
**N1**					
**Adulterants**	**Adulteration level (%)**	**pH**	**Free acidity (mEq Kg^{-1})**	**Conductivity (µs cm^{-1})**	**Moisture content (%)**
**Corn syrup**	0	5.62 ± 0.11	20.24 ± 2.65	981.30 ± 44.83	13.03 ± 1.13
	5	5.62 ± 0.14	18.25 ± 2.80	1007.50 ± 31.82	13.01 ± 0.24
	10	5.65 ± 0.16	18.16 ± 2.66	1014.55 ± 31.75	13.01 ± 1.32
	15	5.68 ± 0.13	17.05 ± 2.66	1022.50 ± 26.16	13.76 ± 0.46
	25	5.69 ± 0.08	15.76 ± 1.20	1029.5 ± 26.16	13.14 ± 0.35
	35	5.74 ± 0.00	11.87 ± 0.11	1035.00 ± 28.28	13.13 ± 1.44
**Date syrup**	5	5.66 ± 0.11	21.01 ± 0.59	1159.00 ± 36.77	13.95 ± 2.21
	10	5.70 ± 0.10	23.34 ± 2.25	1294.50 ± 45.96	13.34 ± 1.20
	15	5.73 ± 0.10	25.02 ± 1.99	1448.50 ± 45.96	14.34 ± 2.48
	25	5.77 ± 0.08	28.76 ± 2.64	1749.00 ± 42.43	14.36 ± 3.15
	35	5.82 ± 0.10	32.69 ± 2.00	2055.00 ± 33.94	14.43 ± 2.97
**Agave syrup**	5	5.96 ± 0.33	17.23 ± 2.12	951.15 ± 43.77	13.70 ± 2.41
	10	6.00 ± 0.28	14.52 ± 1.45	896.45 ± 39.53	13.80 ± 0.37
	15	6.02 ± 1.05	13.39 ± 1.45	859.25 ± 36.56	13.85 ± 0.58
	25	6.02 ± 0.17	12.50 ± 0.26	763.70 ± 31.11	13.99 ± 1.22
	35	6.03 ± 0.13	11.90 ± 0.40	696.80 ± 33.52	14.84 ± 0.07

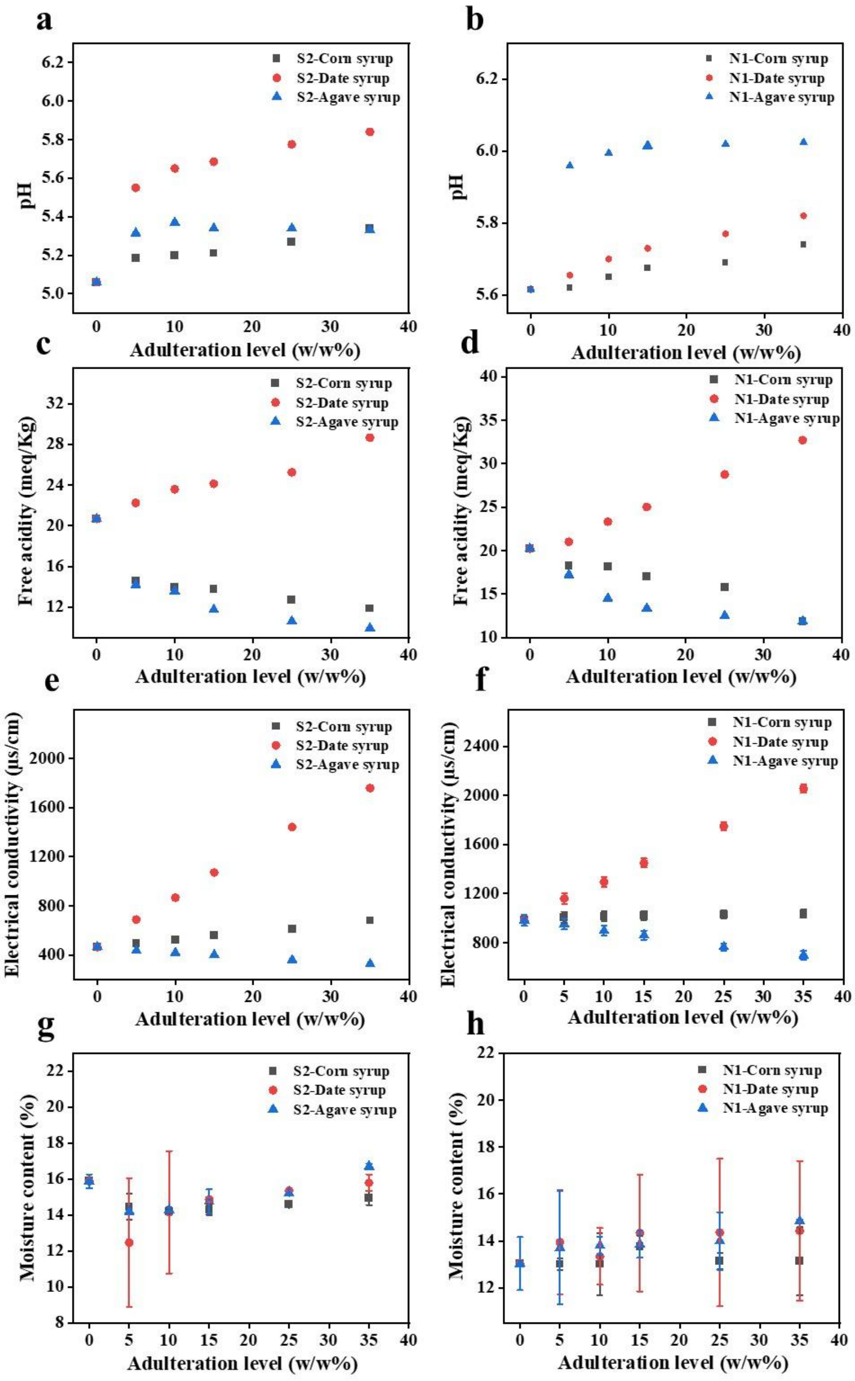

Figure S3. Evaluation of physicochemical parameters in **S2** and **N1** Kuwaiti Sidr honey samples adulterated with corn, date, and agave syrups: **(a-b)** pH, **(c-d)** free acidity, **(e-f)** electrical conductivity and **(g-h)** moisture content. Error bars represent one standard deviation.

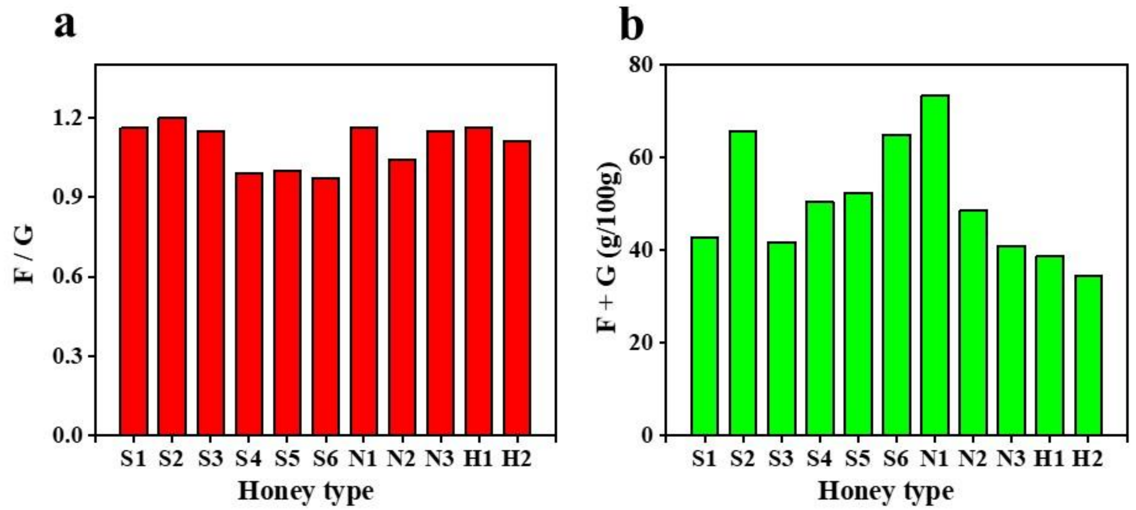

Figure S4. (a) F/G ratio and (b) F+G (g/100 g) for various Kuwaiti Sidr honey samples analyzed using LC–MS.

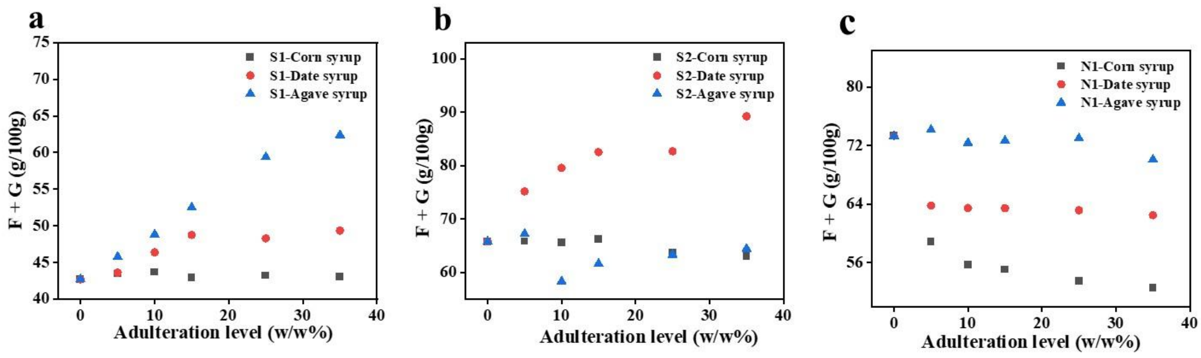

Figure S5. Total sugar content of three different Kuwaiti sider honey samples (**a**) S1 (**b**) S2 and (**c**) N1 adulterated with corn, date and agave syrups.

Table S3. One-way ANOVA results for the effects of corn, date, and agave syrup adulteration on honey physicochemical parameters and sugar composition. Reported statistics include F-values, exact p-values, and effect sizes (η^2).

Adulterant	Properties	F	p-value	Effect size (η^2)
**Corn**	pH	3.1	0.0543	0.3111
	Free acidity	3.52	0.0339	0.3455
	Conductivity	852.48	<0.0001	0.9922
	Moisture content	0.70	0.5618	0.0953
	Fructose	8.16	0.000962	0.5502
	Glucose	7.17	0.0019	0.5183
	F/G ratio	6.92	0.0022	0.5093
	F+G	50.99	<0.0001	0.8845
**Date**	pH	2.94	0.0576	0.3067
	Free acidity	3.04	0.0529	0.3130
	Conductivity	22.61	<0.0001	0.7723
	Moisture content	0.17	0.9165	0.0246
	Fructose	19.45	<0.0001	0.7447
	Glucose	9.78	0.000351	0.5947
	F/G ratio	6.66	0.0027	0.4997
	F+G	71.09	<0.0001	0.9143
**Agave**	pH	2.95	0.0573	0.3071
	Free acidity	2.16	0.1241	0.2450
	Conductivity	147.88	<0.0001	0.9568
	Moisture content	0.07	0.9737	0.0108
	Fructose	16.57	<0.0001	0.7131
	Glucose	3.96	0.0228	0.3727
	F/G ratio	0.48	0.6296	0.0598
	F+G	26.95	<0.0001	0.7823
